# Ultra-processed food intake and impairment across multiple cognitive domains in nationally representative older U.S. adults

**DOI:** 10.3389/fpubh.2025.1695540

**Published:** 2026-01-14

**Authors:** Heejin Lee, Elizabeth Ludwig-Borycz, Claire T. McEvoy, Euridice Martínez-Steele, Neha Khandpur, Steven G. Heeringa, Lindsay H. Ryan, Kenneth M. Langa, Julia A. Wolfson, Cindy W. Leung

**Affiliations:** 1 Department of Nutrition, Harvard T.H. Chan School of Public Health, Boston, MA, United States; 2 Center for Global Health Equity, University of Michigan, Ann Arbor, MI, United States; 3 Centre for Public Health, Institute for Global Food Security, Queen’s University Belfast, Belfast, Northern Ireland, United Kingdom; 4 Department of Nutrition, School of Public Health, University of São Paulo, São Paulo, Brazil; 5 Center for Epidemiological Studies in Health and Nutrition, University of São Paulo, São Paulo, Brazil; 6 Division of Human Nutrition and Health, Wageningen University, Wageningen, Netherlands; 7 Institute for Social Research, University of Michigan, Ann Arbor, MI, United States; 8 Department of Internal Medicine, University of Michigan Medical School, Ann Arbor, MI, United States; 9 Department of Health Policy and Management, Johns Hopkins Bloomberg School of Public Health, Baltimore, MD, United States; 10 Department of International Health, Johns Hopkins Bloomberg School of Public Health, Baltimore, MD, United States

**Keywords:** ultra-processed food, cognitive impairment, older adults, Health and Retirement Study, cognitive domain, executive function

## Abstract

**Introduction:**

Ultra-processed food (UPF) consumption, which accounts for more than 50% of energy intake in the U.S., has steadily increased among older adults over the past decade. UPF consumption is associated with overall cognitive decline, but few studies have examined the associations between UPF consumption and individual cognitive domains. In this study, we examined associations between UPF consumption and impairment in executive function, memory, language, visuospatial, and orientation using data from the longitudinal and nationally representative Health and Retirement Study (HRS).

**Methods:**

Data were drawn from HRS participants who took part in both the 2013 Health Care and Nutrition Study (HCNS) and the 2016 Harmonized Cognitive Assessment Protocol (HCAP) and who did not have dementia or memory problems at baseline (2012) (*n* = 1,408). Dietary intake was assessed using the food frequency questionnaire from 2013 HCNS. UPF was classified using the Nova categorization. The percentage of energy intake from UPF was grouped into sex-specific quintiles (Q). Cognitive impairment was assessed from the 2016 HCAP based on cognitive domain scores, defined as >1.5 SDs below the mean or a T-score = 35. Weighted multivariate-logistic regression was used to estimate the associations between UPF consumption and cognitive outcomes.

**Results:**

In the analytic sample, mean UPF intake was 42.3% energy/day. After adjustment for baseline sociodemographic and health characteristics, higher UPF consumption showed a marginally significant trend toward greater impairment in executive function (Q4 vs. Q1, OR 2.08, 95% CI 1.16–3.74; Q5 vs. Q1, OR 1.73, 95% CI 0.97–3.09; P-trend = 0.052). UPF consumption was not significantly associated with impairment in other cognitive domains.

**Discussion:**

Our findings highlight a potential association between high UPF intake and executive functioning impairment among older U.S. adults.

## 1 Introduction

The number of Americans aged 65 years or older with Alzheimer’s disease and related dementias (ADRD) is expected to reach 14 million by 2060 (1), with associated patient care costs projected to reach $1 trillion by 2050 (2). Accelerated cognitive decline has been reported to occur several years before the diagnosis of ADRD, with timing of decline differing across cognitive domains (3). This underscores the importance of preventive strategies targeting modifiable risk factors in the preclinical stage of dementia.

In recent decades, ultra-processed foods (UPF) have been increasingly produced and consumed in the global food system (4). UPFs are industrially produced foods and beverages that contain additives to enhance palatability, shelf life, and cost-effectiveness, and are typically made with minimal or no intact whole-food ingredients (4, 5). Examples include sugar-sweetened beverages, breakfast cereals, cookies, pizza, and other shelf-stable or convenience foods. Among adults in the United States (U.S.), more than half of energy intake comes from UPFs (6, 7). Intake of UPFs is typically lower in older populations, however amount of UPF consumption has been steadily increasing among U.S. older adults, reaching 57% of total energy intake in 2017–2018 (8). Notably, the increase in UPF consumption in older adults coincides with a decline in the consumption of minimally processed foods, such as fruits and vegetables (8).

This is particularly concerning given that UPFs are energy-dense, high in added sugars, saturated fat, and sodium, and low in dietary fiber (5). In addition, increased consumption of UPFs displaces the consumption of nutrient-dense foods, further contributing to lower overall dietary quality. Poor diet quality has been linked to accelerated cognitive aging and decline (9–11). The increasing consumption of UPFs among older adults raises concerns about potential effects on cognitive function.

Research has suggested that UPF consumption is associated with higher risk of all-cause dementia, and Alzheimer’s disease (12, 13). However, little is known about the associations between UPF consumption and individual cognitive domains such as memory, executive functioning, and language. One prospective cohort study found that higher UPF consumption was associated with faster decline in executive function among Brazilian adults (14). Understanding domain-specific associations is important because the onset and rate of decline differ across cognitive domains, with potential clinical implications. However, evidence from U.S. populations on temporal associations between UPF consumption and individual cognitive domains is limited.

To address this gap, we aimed to examine the associations between UPF consumption and impairment in executive function, memory, language, visuospatial, and orientation among older adults in the U.S. using the nationally representative longitudinal Health and Retirement Study (HRS). Given potential sex differences in food preferences and portion sizes (15, 16), we additionally examined whether the associations between UPF consumption and cognitive impairment differed by sex.

## 2 Methods

### 2.1 Study participants

Data were obtained from the HRS, a nationally representative longitudinal survey of U.S. adults, which includes individuals aged 50 years or older and their spouses or partners. The HRS began recruiting participants in 1992 and surveys conducted on a biennial basis (HRS Core), adding new cohorts every 6 years (NIA U01AG009740, NIA R01AG073289). In addition to the regular biennial Core surveys, the HRS administers supplemental off-year surveys to subsamples, focusing in greater depth on specific topics not covered in the HRS Core surveys. For example, the HRS conducted the Health Care and Nutrition Study (HCNS) in 2013 to collect information on healthcare access, food purchases, food consumption, and dietary intake. Another supplemental survey, the Harmonized Cognitive Assessment Protocol (HCAP), was conducted in 2016 to assess cognitive and neuropsychological performance in detail, with the aim of better understanding cognitive function among older adults aged 65 years or older (NIA R01AG05114). The HRS protocol was approved by the University of Michigan Institutional Review Board, and all participants gave their verbal or written informed consent.

In our analysis, participants who took part in both 2013 HCNS and the 2016 HCAP were included (*n* = 1,609). The analytic sample included participants who had completed a food frequency questionnaire (FFQ), had information on cognitive domain scores, and had no reported history of Alzheimer’s disease, dementia, or memory problem in 2012. We also excluded participants with implausible energy intake (male: <800 kcal/day or >4,000 kcal/day; female: <500 kcal/day or >3,500 kcal/day), or missing covariate information. After this exclusion, 1,408 participants were included in the analytic sample (Supplementary Figure S1).

### 2.2 Dietary assessment and ultra-processed food classification

Dietary intake was assessed using the validated, self-administered FFQ in 2013 HCNS, capturing dietary intake over the past year (17). The Harvard food composition tables were used to estimate total energy and nutrient intake, as well as nutrient intake by food item. We used the Nova classification system to define UPFs. The Nova system categorizes foods into four groups based on the extent and purpose of industrial processing: (1) unprocessed or minimally processed foods, (2) processed culinary ingredients, (3) processed foods, and (4) UPFs (5). Food items from the 2013 HCNS FFQ were classified according to the UPF categorization scheme used in the Nurses’ Health Study and the Health Professionals Follow-up Study, with minor modifications (e.g., classifying hamburgers and chicken/turkey sandwiches as UPFs) (18). The UPF category includes the following food groups: (1) whole grains, (2) grains and derivatives, (3) dairy, (4) fats and oils, (5) processed meats, (6) processed fish, (7) snacks and sweets, (8) sweeteners, (9) sugar-sweetened beverages, (10) other beverages, (11) liquor, (12) sauces, (13) mixed dishes (Supplementary Table S1). A list of unprocessed and minimally processed food items is presented in Supplementary Table S2. We calculated the percentage of total energy intake from UPFs (%EI) and categorized participants into sex-specific quintiles (Q) for analysis. We also calculated energy-adjusted UPF intake (g/day) using the residual method and categorized it into sex-specific quintiles, for sensitivity analyses. Similarly, intakes of unprocessed or minimally processed foods (%EI) were categorized into sex-specific quintiles.

### 2.3 Ascertainment of cognitive impairment

All cognitive outcomes were assessed in the 2016 HCAP, a comprehensive battery of cognitive assessments (19). The assessment battery included Mini-Mental State Examination (MMSE), selected items from the Telephone Interview for Cognitive Status (TICS), Consortium to Establish a Registry for Alzheimer’s Disease (CERAD) immediate and delayed word list learning and recall, retrieval fluency test, letter cancellation test, backward counting, selected items from the 10/66 protocol, immediate and delayed story memory of Brave Man and Wechsler Memory Scale (WMS-IV) logical memory, CERAD word list recognition, immediate and delayed CERAD construction praxis, Symbol-Digit Modalities Test (SDMT), number series, Raven’s standard progressive matrices, trail making test, Center for Epidemiologic Studies Depression Scale (CES-D) depressive symptoms, and an optional smell test (19).

Based on prior confirmatory factor analysis (CFA) of the HRS HCAP neuropsychological battery, we included five cognitive domains: orientation, delayed and recognition memory, executive functioning, language/fluency, and visuospatial ability, as this structure has been shown to demonstrate good model fit for summarizing cognitive performance (20). We utilized Bayesian plausible values (PVs) available from the 2016 HCAP dataset as factor score estimates for cognitive domains of executive function, memory, and language/fluency. Factor score estimates for visuospatial were drawn from the HCAP dataset, where they had been imputed, standardized, and adjusted with age, sex, race and ethnicity, and educational attainment, while scores for orientation was singly imputed (21). All these values were standardized to a mean of 50 and standard deviation of 10 and were then used as continuous outcomes to examine linear associations between UPF consumption and cognitive performance in our analysis. In addition, we used binary outcomes for domain-specific cognitive impairment. Impairment in executive function, memory, language/fluency, and visuospatial ability were provided in the HCAP dataset, where it was defined as standardized score >1.5 SDs below the mean (T-score of 35) consistent with criteria used in dementia algorithms (22). For orientation, a singly imputed score of ≤8 (out of 10) was considered indicative of orientation impairment (21). In the present analysis, we used these domain-level impairment indicators directly.

### 2.4 Covariates

The HRS Core collected information on demographic, socioeconomic, lifestyle, and health-related covariates through its biennial interview questionnaires. To preserve temporality of the variables, we used covariate data from the 2012 HRS core survey; if 2012 covariate data were unavailable, we carried forward data from an earlier wave. The following covariates were selected based on their potential to act as confounding factors for UPF consumption and cognitive impairment, identified by examining ≥7% changes in the effect estimates when each variable was added to the age-, sex-, and race/ethnicity-adjusted model. The covariates included age (years); sex (male, female); race and ethnicity (White, Black, Hispanic, Other: American Indian, Alaskan Native, Asian, and Pacific Islander); marital status (never married, married but spouse absent, separated, divorced, widowed; married or living with a partner); education level (less than high school, high school graduate, some college/college graduate, post-college); total net worth (tertile); household size (1, 2, ≥3 members); vigorous physical activity (no, ≤once/week, >once/week); smoking status (never, past, current smoker); alcohol consumption (0, <5, ≥5 g/day); total energy intake (kcal/day); body mass index (BMI, <25, 25–30, ≥30 kg/m^2^); and depressive symptoms in 2012 (yes, no). Multicollinearity among covariates was assessed using variance inflation factors (VIFs); all values were below 2.5, indicating no concerning collinearity (Supplementary Table S3). In the analytic sample (*n* = 1,408), the proportions of participants with carried-forward covariate values were minimal, with 0.14% for marital status, 0.07% for total net worth, 0.07% for household size, 0.28% for vigorous physical activity, 0.07% for smoking status, and 0.40% for depressive symptoms. Total net worth was obtained by adding all wealth components and subtracting debts (23). Since total net worth was negative (i.e., indicating debt) for some participants, we added a small random constant to all values to make them positive, and then applied a logarithmic transformation (24). The transformed values were then divided into tertiles: low (tertile 1; <$107,900), medium (tertile 2; $107,900–$434,700), and high (tertile 3; >$434,700). Depressive symptoms were assessed using the 8-item version of the Center for Epidemiologic Studies Depression Scale (CESD-8). CESD-8 includes questions about the following symptoms experienced during the past week: feeling depressed, feeling that everything was an effort, having restless sleep, feeling happy, feeling lonely, enjoying life, feeling sad, and feeling unmotivated. A scores ≥5 was used to indicate the presence of depressive symptoms (25).

### 2.5 Statistical analysis

The HRS employs a stratified, multistage area probability sampling design. To account for the complex HRS design and the analytic sample limited to 2013 HCNS and 2016 HCAP participants, we created propensity-based weights (26, 27). We used logistic regression to estimate propensity scores reflecting the likelihood of having HCNS data among HCAP participants adjusting for age, sex, race and ethnicity, and years of education. To avoid extreme weights, we grouped participants into deciles of the estimated propensity scores and computed the inverse of the mean score within each decile. These reciprocals were multiplied by the 2016 HCAP weights and used as attrition-adjusted sampling weights for the analysis and were not further rescaled.

Participants’ characteristics according to UPF consumption were presented as weighted means and standard errors for continuous variables, and as frequencies and weighted percentages for categorical variables. The percentage contribution of each UPF category to total UPF energy intake was calculated, and the top five categories were identified. We examined the associations between UPF consumption and cognitive domain scores using weighted multivariable-adjusted linear regression, reporting *β* estimates with 95% confidence intervals (CIs). Least-squares (LS) means of cognitive domain scores were also estimated across categories of UPF consumption. Linear trends in cognitive domain scores were tested by assigning the median UPF consumption of each category and modeling this as a continuous variable. Model 1 was adjusted for participants’ age, sex, race and ethnicity. Model 2 additionally adjusted for marital status, education level, total net worth, household size, vigorous physical activity, smoking status, alcohol consumption, depressive symptoms in 2012 as covariates. Weighted multivariable logistic regression models estimated odds ratios (ORs) and 95% CIs for the associations between UPF consumption and cognitive impairment, adjusted for the same covariates as the linear regression models. Tests for linear trend were conducted by modeling the median UPF consumption in each quintile as a continuous variable. To test a possible non-linear association with executive function impairment, we used restricted cubic spline analysis with four knots after excluding the top 1% of UPF consumption.

To ensure robustness, we conducted several sensitivity analyses. First, we excluded participants with neurological conditions reported between the 2013 HCNS, when UPF intake was assessed, and the 2016 HCAP assessment, when cognitive function was measured, to mitigate potential reverse causation. Neurological conditions included Alzheimer’s disease and related dementias (ADRD), memory problems, Parkinson’s disease, and stroke. ADRD and stroke were identified through both self-reports and informant interviews, whereas memory problems and Parkinson’s disease were reported by informants only. For Parkinson’s disease and stroke, because we did not exclude individuals with these conditions in the main analysis, we additionally excluded not only those who reported them between 2013 and 2016 but also those with a history of these conditions prior to 2013. Second, we further adjusted for total energy intake, and additionally included BMI, which was considered as a potential mediator. Third, because the units of UPF intake can influence the statistical significance of associations with disease outcomes (28), we additionally used an alternative measure of UPF, treating energy-adjusted UPF intake (g/day) as the exposure. Given that attenuation factors have been shown to be larger for energy-adjusted measures than for absolute measures (e.g., kcal or gram weight) in FFQ-based validation studies of UPF (28), we used energy-adjusted gram intake in our analysis. We also estimated the association between unprocessed and minimally processed food intake and impairment in each cognitive domain. Analyses were performed using SAS 9.4 (SAS Institute Inc., Cary, NC, USA), and *p* < 0.05 was considered statistically significant.

## 3 Results

### 3.1 Descriptive characteristics of participants

In the analytic sample of 1,408 adults, the mean age at baseline (2013) was 72.0 years (SD 7.3), 60.2% (*n* = 848) were female, 76.1% (*n* = 1,072) were White, 13.7% (*n* = 193) were Black, and 8.4% (*n* = 118) were Hispanic. The mean total UPF consumption was 42.3% of total energy (SD 12.0%). The mean unprocessed or minimally processed food (Nova1) accounted for 43.7% (SD 11.7%) of total energy. Participants in the top quintile of UPF consumption were more likely to be male, White or Black individuals, never married or previously married but currently without a spouse, have low total net worth, have lower levels of education, be current smokers, be non-drinkers, be physically inactive, have obesity, and report depressive symptoms, compared to those in the bottom quintile (Table 1). Snacks and sweets (29.69%) were the largest contributors to UPF energy intake, followed by grains and derivatives (14.67%), dairy (11.67%), processed meats (11.05%), and sugar-sweetened beverages (9.29%). The ranking of top contributors was also largely similar between men and women, with minor differences in order; liquor appeared in the top 10 for men and other beverages for women (Supplementary Table S4).

**Table 1:** Characteristics of participants according to ultra-processed food (UPF) intake (*n* = 1,408).

Variable	Total	Quintile 1	Quintile 2	Quintile 3	Quintile 4	Quintile 5
No. of participants	1,408	281	282	282	282	281
Age (years)	70.1 ± 0.3	69.8 ± 0.6	69.5 ± 0.5	71.0 ± 0.5	70.7 ± 0.6	69.8 ± 0.6
Sex, female (*n*, %)	848 (56.3%)	169 (59.9%)	170 (56.9%)	170 (54.2%)	170 (56.0%)	169 (54.3%)
Race/ethnicity						
White	1,072 (84.2%)	187 (76.6%)	206 (78.8%)	217 (86.1%)	236 (91.2%)	226 (88.3%)
Black	193 (7.4%)	31 (6.6%)	38 (7.6%)	41 (7.0%)	35 (6.1%)	48 (9.8%)
Hispanic	118 (6.5%)	56 (14.4%)	29 (10.1%)	21 (5.4%)	7 (1.7%)	5 (0.9%)
Other	25 (1.9%)	7 (2.3%)	9 (3.5%)	3 (1.5%)	4 (1.0%)	2 (1.1%)
Marital status (*n*, %)						
Never married^a^	466 (30.0%)	88 (27.0%)	90 (30.8%)	91 (29.2%)	100 (28.9%)	97 (33.9%)
Married or living with a partner	942 (70.0%)	193 (73.0%)	192 (69.2%)	191 (70.8%)	182 (71.2%)	184 (66.1%)
Total net worth (*n*, %)						
Tertile 1	469 (28.9%)	85 (23.2%)	74 (24.9%)	100 (27.7%)	101 (30.2%)	109 (38.7%)
Tertile 2	470 (32.6%)	90 (29.1%)	107 (34.2%)	91 (36.3%)	80 (30.0%)	102 (33.5%)
Tertile 3	469 (38.5%)	106 (47.7%)	101 (40.9%)	91 (36.0%)	101 (39.8%)	70 (27.8%)
Household size (*n*, %)						
1	321 (22.2%)	63 (21.5%)	62 (22.8%)	63 (20.5%)	69 (20.65%)	64 (25.6%)
2	834 (60.0%)	173 (64.2%)	165 (58.7%)	174 (63.4%)	165 (60.7%)	157 (53.5%)
≥3	253 (17.8%)	45 (14.4%)	55 (18.5%)	45 (16.2%)	48 (18.65%)	60 (21.0%)
Education level (*n*, %)						
Less than high school	241 (13.2%)	63 (13.5%)	42 (12.5%)	48 (14.1%)	47 (12.9%)	41 (13.2%)
High school graduate	491 (32.2%)	69 (22.0%)	94 (28.9%)	103 (36.0%)	109 (38.9%)	116 (35.4%)
Some college/college graduate	487 (38.1%)	100 (40.7%)	102 (40.9%)	95 (36.4%)	93 (34.0%)	97 (38.5%)
Post-college	189 (16.5%)	49 (23.8%)	44 (17.7%)	36 (13.6%)	33 (14.3%)	27 (12.9%)
Smoking status (*n*, %)						
Never smoker	646 (47.0%)	131 (50.8%)	130 (45.7%)	134 (48.8%)	139 (48.1%)	112 (41.9%)
Ever smoker	630 (43.9%)	133 (43.8%)	128 (45.0%)	124 (42.2%)	120 (45.6%)	125 (42.4%)
Current smoker	132 (9.1%)	17 (5.4%)	24 (9.3%)	24 (9.0%)	23 (6.3%)	44 (15.7%)
Alcohol consumption (*n*, %)						
0 g/day	580 (38.2%)	99 (30.8%)	102 (33.5%)	121 (37.3%)	111 (38.1%)	147 (51.7%)
<5 g/day	475 (33.7%)	85 (31.7%)	105 (36.1%)	89 (34.1%)	114 (38.4%)	82 (27.7%)
≥5 g/day	353 (28.2%)	97 (37.5%)	75 (30.4%)	72 (28.6%)	57 (23.6%)	52 (20.6%)
Vigorous activity (*n*, %)						
No	548 (36.4%)	69 (21.5%)	101 (30.7%)	108 (36.7%)	122 (41.6%)	148 (51.9%)
≤Once/week	329 (24.4%)	65 (23.4%)	60 (23.3%)	89 (33.9%)	69 (24.0%)	46 (17.6%)
>Once/week	531 (39.2%)	147 (55.2%)	121 (46.0%)	85 (29.5%)	91 (34.4%)	87 (30.5%)
Body mass index (BMI)						
<25 kg/m^2^	281 (18.7%)	65 (23.2%)	60 (17.7%)	50 (17.4%)	50 (16.9%)	56 (18.7%)
25–30 kg/m^2^	493 (36.0%)	105 (36.6%)	104 (40.4%)	95 (33.7%)	100 (36.2%)	89 (33.0%)
≥30 kg/m^2^	634 (45.2%)	111 (40.3%)	118 (42.0%)	137 (48.9%)	132 (46.9%)	136 (48.3%)
Depressive symptoms (*n*, %)^b^	86 (5.3%)	12 (2.3%)	18 (5.3%)	17 (5.49%)	12 (3.79%)	27 (9.78%)
Total energy intake (kcal/d)	1629.4 ± 16.9	1587.0 ± 36.0	1634.3 ± 43.7	1605.7 ± 41.0	1680.4 ± 0.6	1636.8 ± 44.2
UPF intake (%EI)	42.4 ± 0.5	25.9 ± 0.4	36.1 ± 0.2	42.5 ± 0.1	48.6 ± 0.2	59.3 ± 0.6

The percentage of energy intake (%EI) from ultra-processed foods was categorized into sex-specific quintiles.

Characteristics are presented for the analytic samples for dementia. Continuous variables are presented as weighted mean ± standard errors. Categorical variables are presented as numbers and weighted percentages.

^a^Never married, married but spouse absent, separated, divorced, widowed were included in this category. ^b^Depressive symptoms were defined as a Center for Epidemiologic Studies Depression Scale-8 items (CESD-8) score of ≥5 at baseline.

### 3.2 Associations between UPF consumption and cognitive domain score

The associations between UPF consumption and standardized scores of the five cognitive domains are shown in Table 2; a significant association was observed only for executive function. In the age-, sex-, race and ethnicity-adjusted model (Model 1), for the executive function score, the *β* (difference) was −2.75 (95% CI: −4.19, −1.32) for Q4 (compared to Q1) and −2.25 (95% CI −3.88, −0.62) for Q5 (compared to Q1) (P-trend = 0.001). In the fully adjusted model (Model 2), the inverse association between UPF consumption and executive functioning was attenuated, with the association remaining significant only for Q4 (compared to Q1) (*β* = −1.44, 95% CI: −2.76, −0.12; P-trend = 0.395). The LS-means were 46.72 (95% CI: 44.88–48.55) for Q1, 45.28 (95% CI: 43.53–47.04) for Q4, and 46.48 (95% CI: 44.53–48.44) for Q5 in Model 2.

**Table 2:** Multivariate-adjusted associations between percentage energy from ultra-processed food (UPF) intake and cognitive domain scores^a^ (*n* = 1,408).

Estimates	Cognitive domain	Model	Ultra-processed food intake					
			Quintile 1	Quintile 2	Quintile 3	Quintile 4	Quintile 5	P-trend
Median (IQR)^b^			27.7 (22.9–30.2)	36.1 (34.2–38.1)	42.3 (41.0–43.8)	48.4 (46.8–50.1)	56.9 (54.2–61.1)	
*β* (95% CI)^c^	Executive function	Model 1^d^	Reference	−0.78(−2.47, 0.92)	−0.52(−2.04, 1.01)	−2.75(−4.19, −1.32)	−2.25(−3.88, −0.62)	0.001
		Model 2^e^	Reference	−0.23(−1.63, 1.17)	0.74(−0.50, 1.99)	−1.44(−2.76, −0.12)	−0.24(−1.69, 1.21)	0.395
	Memory	Model 1^d^	Reference	−1.15(−3.04, 0.74)	−1.18(−3.25, 0.89)	−1.48(−3.30, 0.35)	−1.30(−3.04, 0.43)	0.111
		Model 2^e^	Reference	−0.53(−2.26, 1.19)	−0.05(−2.12, 2.02)	−0.27(−2.19, 1.64)	0.51(−1.31, 2.33)	0.575
	Language	Model 1^d^	Reference	−1.49(−3.44, 0.46)	−1.31(−3.19, 0.57)	−0.48(−2.57, 1.62)	−1.53(−3.37, 0.30)	0.208
		Model 2^e^	Reference	−0.85(−2.83, 1.13)	−0.16(−1.88, 1.56)	0.98(−1.17, 3.14)	0.45(−1.28, 2.19)	0.227
	Visuospatial	Model 1^d^	Reference	0.31(−1.89, 2.51)	0.74(−1.33, 2.81)	−0.89(−2.88, 1.10)	−0.21(−2.07, 1.65)	0.223
		Model 2^e^	Reference	0.51(−1.63, 2.64)	0.92(−1.29, 3.14)	−0.58(−2.57, 1.41)	0.78(−1.01, 2.66)	0.970
	Orientation	Model 1^d^	Reference	−0.92(−2.84, 1.01)	−0.16(−1.56, 1.24)	−0.72(−3.19, 1.75)	−0.78(−2.70, 1.14)	0.631
		Model 2^e^	Reference	−0.93(−2.71, 0.86)	0.14(−1.21, 1.48)	−0.42(−2.94, 2.09)	−0.39(−2.23, 1.44)	0.612
LS-means	Executive function	Model 1^d^	47.82(45.81–49.83)	47.04(45.05–49.04)	47.3(45.46–49.15)	45.06 (43.37–46.76)	45.57 (43.80–47.35)	0.001
		Model 2^e^	46.72 (44.88–48.55)	46.49 (44.48–48.49)	47.46 (45.68–49.24)	45.28 (43.53–47.04)	46.48 (44.53–48.44)	0.395
	Memory	Model 1^d^	48.64 (46.45–50.82)	47.49 (45.2–49.77)	47.46 (45.52–49.40)	47.16 (44.84–49.48)	47.33 (45.17–49.50)	0.111
		Model 2^e^	47.18 (44.90–49.45)	46.64 (44.36–48.92)	47.12 (44.83–49.41)	46.90 (44.35–49.46)	47.68 (45.33–50.04)	0.575
	Language	Model 1^d^	48.34 (46.55–50.13)	46.85 (45.18–48.52)	47.03 (45.19–48.86)	47.87 (45.84–49.89)	46.81 (45.04–48.58)	0.208
		Model 2^e^	48.33 (46.51–50.15)	47.48 (45.60–49.36)	48.17 (45.96–50.38)	49.32 (46.91–51.72)	48.78 (46.98–50.59)	0.227
	Visuospatial	Model 1^d^	50.32 (48.49–52.14)	50.63 (49.02–52.23)	51.05 (49.44–52.67)	49.42 (47.7–51.15)	50.11 (48.27–51.95)	0.223
		Model 2^e^	49.78 (47.66–51.90)	50.29 (48.04–52.54)	50.70 (48.41–53.00)	49.20 (46.79–51.61)	50.56 (48.35–52.78)	0.970
	Orientation	Model 1^d^	47.47 (44.88–50.05)	46.55 (43.79–49.32)	47.31 (44.82–49.79)	46.75 (43.66–49.84)	46.69 (43.69–49.68)	0.631
		Model 2^e^	47.58 (44.97–50.18)	46.65 (43.81–49.49)	47.71 (45.14–50.29)	47.15 (43.82–50.49)	47.18 (44.24–50.13)	0.612

^a^All cognitive domain scores were standardized to a mean of 50 and a standard deviation of 10. ^b^Values are presented as the percentage of energy intake from ultra-processed food, with sex-specific quintile was applied. ^c^*β* represents the difference in cognitive domain score compared with the reference group. ^d^Model 1 was adjusted for age (continuous, years), gender (men, women), and race/ethnicity (White, Black, Hispanic, and other). ^e^Model 2 was further adjusted for marital status (never married, married but spouse absent, separated, divorced, widowed; married or living with a partner), education (less than high school, high school graduate, some college/college graduate, post-college), total net worth (tertile), household size (1, 2, ≥3 members), vigorous activity (no, ≤once/week, >once/week), smoking (never smoker, ever smoker, current smoker), alcohol consumption (nondrinker, <5 g/day, ≥5 g/day), and baseline depressive symptom (yes, no; CESD-8 score ≥5). All models accounted for the complex sampling design.

### 3.3 Associations between UPF consumption and domain-specific cognitive impairment

The associations between UPF consumption and cognitive impairment across five domains are presented in Table 3. UPF consumption was associated with higher odds of executive function impairment (compared to Q1: Q4 OR = 2.19, 95% CI: 1.23–3.87; Q5 OR = 2.03, 95% CI: 1.18–3.50; P-trend = 0.006) in Model 1. In Model 2, greater UPF consumption showed a marginally significant trend toward higher odds of executive function impairment (compared to Q1: Q4 OR = 2.08, 95% CI: 1.16–3.74; Q5 OR = 1.73, 95% CI: 0.97–3.09; P-trend = 0.052). The restricted cubic spline analysis showed no evidence of a linear association between UPF consumption and executive function impairment (P-linearity = 0.248; Supplementary Figure S2). No significant associations were observed between UPF consumption and impairments in memory, language, visuospatial ability, or orientation.

**Table 3:** Multivariate-adjusted associations between percentage of energy intake from ultra-processed food (UPF) and domain-specific cognitive impairment.

Cognitive outcome	Quintile 1	Quintile 2	Quintile 3	Quintile 4	Quintile 5	P-trend
Executive function impairment						
Cases/total	24/281	37/282	23/282	46/282	33/281	
Model 1^a^	1.00	1.63 (0.79–3.34)	0.81 (0.40–1.62)	2.19 (1.23–3.87)	2.03 (1.18–3.50)	0.006
Model 2^b^	1.00	1.64 (0.82–3.30)	0.76 (0.40–1.44)	2.08 (1.16–3.74)	1.73 (0.97–3.09)	0.052
Memory impairment						
Cases/total	27/281	44/282	36/282	33/282	27/281	
Model 1^a^	1.00	1.85 (0.89–3.87)	1.21 (0.55–2.67)	1.10 (0.59–2.07)	0.84 (0.44–1.60)	0.183
Model 2^b^	1.00	1.69 (0.81–3.54)	1.10 (0.50–2.44)	0.96 (0.48–1.92)	0.61 (0.28–1.33)	0.052
Language impairment						
Cases/total	28/281	26/282	38/282	34/282	26/281	
Model 1^a^	1.00	1.13 (0.46–2.75)	2.26 (1.13–4.56)	1.47 (0.74–2.93)	1.64 (0.81–3.35)	0.159
Model 2^b^	1.00	1.04 (0.41–2.66)	2.12 (0.996–4.52)	1.37 (0.67–2.80)	1.18 (0.54–2.59)	0.579
Visuospatial impairment						
Cases/total	23/281	29/282	30/282	27/282	22/281	
Model 1^a^	1.00	1.21 (0.56–2.61)	1.01 (0.42–2.43)	0.94 (0.41–2.16)	0.79 (0.37–1.66)	0.433
Model 2^b^	1.00	1.20 (0.58–2.47)	1.09 (0.43–2.80)	0.98 (0.42–2.27)	0.70 (0.30–1.66)	0.366
Orientation impairment						
Cases/total	29/281	34/282	31/282	26/282	31/281	
Model 1^a^	1.00	1.54 (0.76–3.13)	1.11 (0.66–1.85)	1.30 (0.54–3.11)	2.22 (1.05–4.71)	0.066
Model 2^b^	1.00	1.70 (0.85–3.37)	1.05 (0.61–1.80)	1.18 (0.49–2.82)	2.11 (0.96–4.65)	0.159

Sample size (*n*) = 1,408. Estimates are presented as odds ratios (ORs) and corresponding 95% confidence intervals (CIs).

Ranges for UPF intake (% EI) were defined using sex-specific quintiles, which resulted in overlapping ranges across adjacent quintiles in the total sample. For the total sample: Q1 = 2.7–32.6%, Q2 = 31.7–40.1%, Q3 = 38.8–46.2%, Q4 = 44.7–53.5%, Q5 = 51.1–93.9%. For men: Q1 = 11.4–32.6%, Q2 = 32.8–40.1%, Q3 = 40.1–46.2%, Q4 = 46.4–53.5%, Q5 = 53.5–86.6%. For women: Q1 = 2.7–31.7%, Q2 = 31.7–38.8%, Q3 = 38.8–44.7%, Q4 = 44.7–51.1%, Q5 = 51.1–93.9%.

^a^Model 1 was adjusted for age (continuous, years), gender (men, women), and race/ethnicity (White, Black, Hispanic, and other).

^b^Model 2 was further adjusted for marital status (never married, married but spouse absent, separated, divorced, widowed; married or living with a partner), education (less than high school, high school graduate, some college/college graduate, post-college), total net worth (tertile), household size (1, 2, ≥3 members), vigorous activity (no, ≤once/week, >once/week), smoking (never smoker, ever smoker, current smoker), alcohol consumption (nondrinker, <5 g/day, ≥5 g/day), and baseline depressive symptom (yes, no; CESD-8 score ≥5). All models accounted for the complex sampling design. All models accounted for the complex sampling design.

In sensitivity analyses, we excluded participants who had neurological conditions such as memory problems, stroke, or Parkinson’s disease reported prior to the 2016 HCAP interview (*n* = 248). These neurological conditions occurred after ultra-processed food intake was measured in 2013 but before cognitive impairment was assessed in 2016. The overall associations remained similar; however, the association between ultra-processed food intake and executive function impairment became more pronounced (Table 4). Compared to the lowest quintile of UPF consumption (Q1), Q4 (OR = 2.41, 95% CI: 1.07–5.44) and Q5 (OR = 2.50, 95% CI: 1.02–6.08) were significantly associated with a higher prevalence of executive function impairment in the fully adjusted model (P-trend = 0.022). Furthermore, after controlling total energy intake and BMI, the associations were consistent, with a more pronounced association for executive function impairment (Supplementary Table S5). When UPF intake was modeled as energy-adjusted grams in quintiles, the associations appeared stronger in Q3 (OR = 2.75, 95% CI: 1.29–5.85) and Q5 (OR = 2.25, 95% CI: 1.06–4.80), while the association in Q4 attenuated (P-trend = 0.172) (Supplementary Table S6). Additionally, energy-adjusted UPF consumption was significantly associated with higher odds of orientation impairment (Q5 vs. Q1, OR = 2.23; 95% CI: 1.10–4.52; P-trend = 0.021).

**Table 4:** Multivariate-adjusted associations between percentage of energy intake from ultra-processed food (UPF) and domain-specific cognitive impairment, after excluding participants with neurological conditions reported prior to the 2016 HCAP interview^a^.

Cognitive outcome	Quintile 1	Quintile 2	Quintile 3	Quintile 4	Quintile 5	P-trend
Executive function impairment						
Cases/total	16/231	19/232	13/233	27/232	24/232	
Model 1^b^	1.00	1.46 (0.63–3.40)	0.83 (0.30–2.30)	2.46 (1.12–5.39)	2.78 (1.30–5.94)	0.004
Model 2^c^	1.00	1.52 (0.63–3.64)	0.81 (0.30–2.19)	2.41 (1.07–5.44)	2.50 (1.02–6.08)	0.022
Memory impairment						
Cases/total	17/231	24/232	23/233	19/232	17/232	
Model 1^b^	1.00	1.28 (0.55–2.96)	1.34 (0.49–3.68)	1.09 (0.45–2.67)	0.92 (0.41–2.02)	0.680
Model 2^c^	1.00	1.08 (0.46–2.56)	1.13 (0.43–2.98)	0.88 (0.35–2.20)	0.58 (0.24–1.43)	0.150
Language impairment						
Cases/total	22/231	11/232	23/233	21/232	21/232	
Model 1^b^	1.00	0.93 (0.36–2.43)	1.99 (0.88–4.49)	1.49 (0.69–3.18)	1.86 (0.90–3.84)	0.056
Model 2^c^	1.00	0.77 (0.30–1.98)	1.60 (0.68–3.77)	1.30 (0.61–2.75)	1.27 (0.56–2.90)	0.314
Visuospatial impairment						
Cases/total	14/231	20/232	20/233	17/232	16/232	
Model 1^b^	1.00	1.23 (0.51–2.98)	0.97 (0.35–2.71)	0.97 (0.39–2.43)	0.89 (0.30–2.62)	0.718
Model 2^c^	1.00	1.20 (0.49–2.93)	1.13 (0.37–3.44)	1.09 (0.42–2.80)	0.82 (0.23–2.90)	0.709
Orientation impairment						
Cases/total	23/231	15/232	19/233	14/232	19/232	
Model 1^b^	1.00	0.88 (0.38–2.08)	0.76 (0.34–1.69)	1.12 (0.40–3.14)	1.95 (0.78–4.86)	0.147
Model 2^c^	1.00	0.98 (0.39–2.44)	0.75 (0.30–1.91)	1.04 (0.34–3.23)	1.88 (0.68–5.15)	0.232

Estimates are presented as odds ratios (ORs) and corresponding 95% confidence intervals (CIs).

UPF consumption was categorized as sex-specific quintiles.

^a^Neurological conditions include dementia, memory problems, stroke, and Parkinson’s disease. The conditions were identified based on self-reported diagnoses from the 2014 and 2016 HRS Core interviews and informant-reported diagnoses from the 2016 HCAP informant interview. Specifically, Alzheimer’s disease and related dementias (ADRD) were ascertained from both self-reports and informant interviews; memory problems and Parkinson’s disease were reported by informants; and stroke diagnoses were obtained from both self-reports and informant interviews.

^b^Model 1 was adjusted for age (continuous, years), gender (men, women), and race/ethnicity (White, Black, Hispanic, and other).

^c^Model 2 was further adjusted for marital status (never married, married but spouse absent, separated, divorced, widowed; married or living with a partner), education (less than high school, high school graduate, some college/college graduate, post-college), total net worth (tertile), household size (1, 2, ≥3 members), vigorous activity (no, ≤once/week, >once/week), smoking (never smoker, ever smoker, current smoker), alcohol consumption (nondrinker, <5 g/day, ≥5 g/day), and baseline depressive symptom (yes, no; CESD-8 score ≥5). All models accounted for the complex sampling design.

We also examined associations with unprocessed or minimally processed food (Supplementary Table S7). Compared to the lowest quintile (Q1), participants in the highest quintile (Q5) had 58% lower odds of executive function impairment (95% CI: 0.21–0.84; P-trend = 0.013). No significant associations were observed for the other cognitive domains.

### 3.4 Subgroup analysis

To examine potential sex differences, we conducted subgroup analyses stratified by sex (Table 5). In women, greater UPF consumption was associated with higher odds for language impairment (Q5 vs. Q1 OR = 2.63, 95% CI: 0.86–8.05; P-trend = 0.02), whereas no statistically significant association was observed in men (P-interaction = 0.019). For executive function impairment and other cognitive domains, including visuospatial ability and orientation, associations were not different across men and women.

**Table 5:** Multivariate-adjusted associations between percentage of energy intake from ultra-processed food (UPF) and domain-specific cognitive impairment stratified by gender.

Outcome (by gender)	Cases/total	Quintile 1	Quintile 2	Quintile 3	Quintile 4	Quintile 5	P-trend	P-interaction
Executive function impairment								
Men	62/560	1.00	1.27 (0.38–4.28)	0.52 (0.14–1.92)	1.80 (0.59–5.48)	0.85 (0.25–2.90)	0.931	0.553
Women	101/848	1.00	2.03 (0.84–4.92)	1.07 (0.45–2.54)	2.28 (1.03–5.05)	2.73 (1.07–6.95)	0.041	
Memory impairment								
Men	63/560	1.00	1.55 (0.49–4.91)	1.21 (0.37–4.04)	0.61 (0.20–1.90)	0.45 (0.14–1.52)	0.035	0.475
Women	104/848	1.00	1.81 (0.73–4.50)	1.04 (0.41–2.63)	1.17 (0.45–3.03)	0.76 (0.23–2.54)	0.429	
Language impairment								
Men	72/560	1.00	0.99 (0.35–2.83)	1.00 (0.39–2.56)	0.74 (0.28–2.01)	0.52 (0.15–1.80)	0.234	0.019
Women	80/848	1.00	0.74 (0.22–2.46)	4.39 (1.84–10.43)	2.30 (0.96–5.51)	2.63 (0.86–8.05)	0.020	
Visuospatial impairment								
Men	52/560	1.00	1.24 (0.33–4.64)	0.83 (0.23–3.01)	1.25 (0.34–4.56)	0.56 (0.18–1.79)	0.334	0.890
Women	79/848	1.00	1.26 (0.56–2.83)	1.22 (0.42–3.55)	0.80 (0.28–2.31)	0.73 (0.22–2.46)	0.443	
Orientation impairment								
Men	62/560	1.00	2.11 (0.59–7.55)	1.99 (0.64–6.16)	1.37 (0.41–4.61)	4.71 (1.20–18.54)	0.070	0.249
Women	89/848	1.00	1.42 (0.58–3.47)	0.72 (0.30–1.77)	1.04 (0.34–3.23)	1.27 (0.49–3.26)	0.875	

Estimates are presented as odds ratios (ORs) and corresponding 95% confidence intervals (CIs).

UPF consumption was categorized as sex-specific quintiles.

Models were adjusted for age (continuous, years), race/ethnicity (White, Black, Hispanic, and other), marital status (never married, married but spouse absent, separated, divorced, widowed; married or living with a partner), education (less than high school, high school graduate, some college/college graduate, post-college), total net worth (tertile), household size (1, 2, ≥3 members), vigorous activity (no, ≤once/week, >once/week), smoking (never smoker, ever smoker, current smoker), alcohol consumption (nondrinker, <5 g/day, ≥5 g/day), and baseline depressive symptom (yes, no; CESD-8 score ≥5).

## 4 Discussion

This nationally representative study evaluated the associations between UPF consumption and impairment in the cognitive domains of executive function, memory, language, visuospatial, and orientation among older U.S. adults participating in the HRS. We found a marginally significant association between higher UPF consumption and worse executive function, as well as higher odds of executive function impairment. We also found that greater consumption of unprocessed or minimally processed foods was associated with lower odds of executive function impairment. No significant associations were found between UPF consumption and the other cognitive domains.

Our findings of UPF consumption being associated with cognitive impairment are supported by prior findings. Yet, few studies have specifically examined its associations with distinct cognitive domains. For example, one study using data from the UK Biobank did not find differences in prospective memory, reasoning score, and reaction time by UPF consumption level (29). Although the cognitive domains are not fully overlapping across studies, our study also did not observe associations for memory, language, visuospatial, or orientation. Notably, a cross-sectional analysis of NHANES 2011–2014 found an inverted U-shaped association with UPF consumption and verbal fluency as a component of executive function and working memory, with UPF intake peaking at approximately 40–63% of total energy intake (30). Verbal fluency in NHANES was assessed using the animal naming test (30), whereas in HCAP the animal naming task was incorporated into the language domain composite that also included reading and writing tasks (20), which may partly explain our null findings for language impairment. Overall, our study aligns with these findings as well as those from the Brazilian Longitudinal Study of Adult Health, which showed that UPF consumption exceeding 19.9% of total daily energy intake was associated with a faster rate of global and executive function decline (14).

Given that executive function tends to decline earlier than other cognitive domains in older age (31, 32), the association we observed with executive function but not with other domains may be partially explained by the relatively short interval between dietary and cognitive assessments in our study. Executive function decline may also precede and contribute to subsequent impairments in memory and behavior, both of which can be influenced by dietary factors. A meta-analysis of randomized controlled trials found that healthy dietary patterns, such as the Mediterranean or low-fat diet (33), often contrasted with UPF-rich diets, improved executive function but showed no effect on delayed memory, over follow-up periods ranging from several months to 8 years (32).

Our findings are further supported by evidence on UPF and dementia. A prospective cohort study using data from the UK Biobank found that UPF consumption was associated with a higher risk of all-cause dementia and vascular dementia (29). In the Framingham Heart Study, higher UPF consumption among individuals younger than 68 years was associated with an increased risk of Alzheimer’s disease (13). While one U.S. study did not observe a significant association between daily UPF servings and cognitive impairment, including both dementia and cognitive impairment without dementia (CIND) (34), a meta-analysis of observational studies, most of which were prospective cohorts, found that higher UPF intake was associated with a 44% increased risk of incident dementia (12).

Although the underlying biological mechanisms have not been fully elucidated, it is plausible that factors such as gut microbiota composition and lower dietary quality, such as lower intake of B vitamins and omega-3 fatty acids, may partly account for the observed associations with executive function impairment. Omega-3 fatty acids and B vitamins have been suggested to act synergistically to slow cognitive decline (35), and dietary docosahexaenoic acid (an omega-3 fatty acid) has been shown to activate the dorsolateral prefrontal cortex (DLPFC) (36), a subregion of the prefrontal cortex (PFC) involved in executive function and dietary self-control (37, 38). Impaired DLPFC activity may reduce inhibitory control over impulsive eating (38, 39) and may increase susceptibility to highly palatable, energy-dense foods such as UPFs (38–40), suggesting a potential pathway linking UPF consumption and executive function. In addition, synthetic additives commonly present in UPFs, such as dietary emulsifiers and non-nutritive sweeteners, may disrupt intestinal integrity, promote inflammation, and modify bacteriostatic effects (41). Moreover, UPF consumption may alter gut microbiota composition, leading to gut dysbiosis, impaired short-chain fatty acid production, and increased intestinal permeability, changes that could contribute to neuroinflammation, neurodegeneration, and possibly cognitive decline (42, 43). Future studies are needed to better determine the underlying mechanisms.

We observed a significant difference in the associations between UPF intake and language impairment by sex, whereas no significant sex differences were found for executive function or other cognitive domains. Associations with UPF intake were more pronounced among women for language impairment. Although men had slightly higher UPF intake, approximately 2% more energy from UPFs compared to women, this small difference alone is unlikely to explain the observed associations. Further studies are warranted to better understand potential sex differences in the associations between UPF consumption and cognitive impairment.

Unprocessed or minimally processed food consumption was comparable to UPF consumption in our sample, each accounting for approximately 42% of total energy intake. The association between UPF consumption and impaired executive function highlights the potential importance of implementing nutrition strategies to shift dietary intake away from UPFs and promote unprocessed or minimally processed food choices over UPF consumption among older adults. Notably, in our study, a suggestive linear trend was observed between higher UPF intake and the odds of executive function impairment, although the association was not statistically significant among individuals with intake levels below approximately 45% of total energy. A recent meta-analysis also supports the absence of a significant association between moderate UPF intake and dementia risk (12). While reducing UPF intake to very low levels may be challenging in real-world settings, our findings suggest that efforts to reduce UPF consumption, at least to moderate levels, may mitigate potential cognitive risks. Our finding that unprocessed or minimally processed food consumption was associated with lower odds of executive function impairment further supports the potential need to reduce UPF intake. Of note, in the U.S., UPFs account for more than half of the food consumed even at home (43). Particularly among older adults, UPF intake has increased over time for food consumed at home (43). Even among Americans who frequently cook at home, meals often include packaged or ready-to-eat components, meaning UPFs still comprise a large proportion of total energy intake (44). Previous research has shown that limited engagement in home food preparation skills is associated with higher energy intake from UPFs (45), and that more frequent and longer time spent cooking is associated with lower UPF consumption (44). Therefore, rather than simply advising individuals to reduce UPF intake, public health strategies may benefit from encouraging cooking and promoting practical food preparation skills through community-level education initiatives to facilitate healthier eating behaviors. Such programs could also support family members and caregivers who are often involved in meal preparation for older adults. In parallel, improving the availability, accessibility, and affordability of unprocessed and minimally processed foods may further facilitate healthier eating (46). Moreover, policy-level approaches such as front-of-package (FOP) nutrition, UPF warning labels, or the use of UPF markers (e.g., additives or ingredients that indicate ultra-processing) may also help promote healthier food choices (46–48). Although such labels have not yet shown effects on purchase intentions, they appear to improve consumer’s ability to identify UPFs (47). Combining nutrient-based (e.g., high in sugar and saturated fat) and processing-based (e.g., UPF) information may further support healthier food choices (48).

Key strengths of our study include its nationally representative sample of older adults, the use of a validated and widely used FFQ to assess dietary intake, and in-depth cognitive measures from HRS HCAP, which are designed to be comparable with HRS sister studies conducted in countries such as Mexico, India, and England. In addition, several sensitivity analyses confirmed the robustness of our findings. However, this study has several limitations that should be acknowledged. The largest association between UPF consumption and executive function was observed in the fourth quintile of UPF intake, but not significant in the highest quintile of UPF intake. In restricted spline curve analysis, the curve peaked at mid-range UPF intake (approximately 45%) and slightly declined thereafter, although there were no significant linear or non-linear associations. A similar non-linear pattern between UPF consumption and executive function/working memory, with the peak observed at approximately 40–63% of total energy intake, was also reported in a prior analysis using NHANES 2011–2014 data (30), supporting the attenuation in the top quintile. Notably, the average UPF consumption in our sample was lower than estimates from NHANES based on 24-h dietary recalls (8), consistent with prior evidence that FFQs generally underestimate intake compared with 24-h dietary recalls (28, 49). In addition, a reported tendency to avoid foods perceived as UPFs, especially among those with higher education and income (50), may reflect social desirability bias, potentially leading to underreporting of UPF consumption. Such underestimation of UPF intake is likely to attenuate observed associations with cognitive impairment toward the null. Although we accounted for a range of potential confounding factors, unmeasured or residual confounding cannot be ruled out. For example, we did not adjust for family history of cognitive impairment, because it was collected only in the 2010 experimental module, which was administered to approximately 10% of Core participants, limiting its inclusion as a covariate. Since our analytic sample included participants from both the HCNS and HCAP waves, potential selection bias cannot be ruled out. To address this issue, we applied propensity-based weights based on age, sex, race and ethnicity, and education, referencing the HCAP sample as the target population. Although participants with both HCNS and HCAP data may represent a somewhat healthier subset, with a higher proportion reporting vigorous physical activity, socioeconomic and lifestyle characteristics, such as education, marital status, and smoking status, were largely comparable to those of the overall HRS population at the 2012 core survey, suggesting that selection bias is likely minimal. Despite the prospective design of this study, which allows for temporal associations, both dietary intake and cognitive function were only assessed at single time points. This prevents our ability to evaluate changes in dietary intake predicting changes in cognitive function over time and to infer causality. Furthermore, the timing of dietary intake and cognitive measures was spaced 3 years apart. Given that cognitive impairment develops gradually over time (51), future studies with repeated and longer-term dietary and cognitive assessments are warranted to better capture subtle changes in the exposure and outcome and to better clarify the temporal nature of this association. Although the HRS core survey collects cognitive measures biennially, the HCAP provides a more comprehensive cognitive assessment, allowing for the evaluation of individual cognitive domains. Reverse causation cannot be overlooked, as progression of cognitive impairment may interfere with the ability to maintain a healthy diet. To minimize this possibility, we excluded participants who had dementia or self-reported memory problems at baseline. Moreover, the persistence of the association after excluding individuals who developed neurological conditions yielded similar results. Nonetheless, despite these efforts, the possibility of reverse causation cannot be entirely ruled out. Although we used a validated FFQ, it was not originally designed to assess UPF consumption, which may have led to misclassification of UPF intake. However, there is currently no gold standard for measuring UPF intake, which is typically derived from dietary assessment tools such as FFQs or 24-h dietary recalls. In our study, we generally adopted a previous categorization of FFQ items into Nova groups developed for large U.S. cohort studies (including the Nurses’ Health Study, the Health Professionals Follow-up Study, and the Growing Up Today Study), based on a rigorous multi-stage process involving independent coding and expert consensus (18). When UPFs are classified through expert consensus, items with insufficient detail are often classified conservatively as non-UPF, which may further contribute to underestimation of UPF intake (28). Conversely, some food items in the HRS (e.g., yogurt, low-fat, artificially sweetened, or plain) were grouped into a single question rather than being assessed separately and were therefore classified as UPF in our analysis, which may have introduced some degree of non-differential misclassification. Such misclassification could have attenuated true associations, shifting the results toward the null. Replication of our findings in other populations and longitudinal settings with extended follow-up is warranted.

## 5 Conclusion

Among older adults, UPF consumption was marginally associated with higher odds of executive function impairment. Our findings underscore the potential need for public health efforts to reduce UPF consumption in older populations. Future studies are warranted to confirm our findings of the association between UPF consumption and impairment in specific cognitive domains, particularly executive function.

## Data Availability

Publicly available datasets were analyzed in this study. This data can be found here: https://hrs.isr.umich.edu/.
