# Supplementary material for: Ultra-processed food intake and impairment across multiple cognitive domains in nationally representative older U.S. adults
Source: Front Public Health. 2026 Jan 14;13:1695540. doi: 10.3389/fpubh.2025.1695540 (PMC12850515; doi:10.3389/fpubh.2025.1695540)
Supplement: Supplementary file 1 [file Data_Sheet_1.pdf]

# *Supplementary Material*

## 1 Supplementary Figures

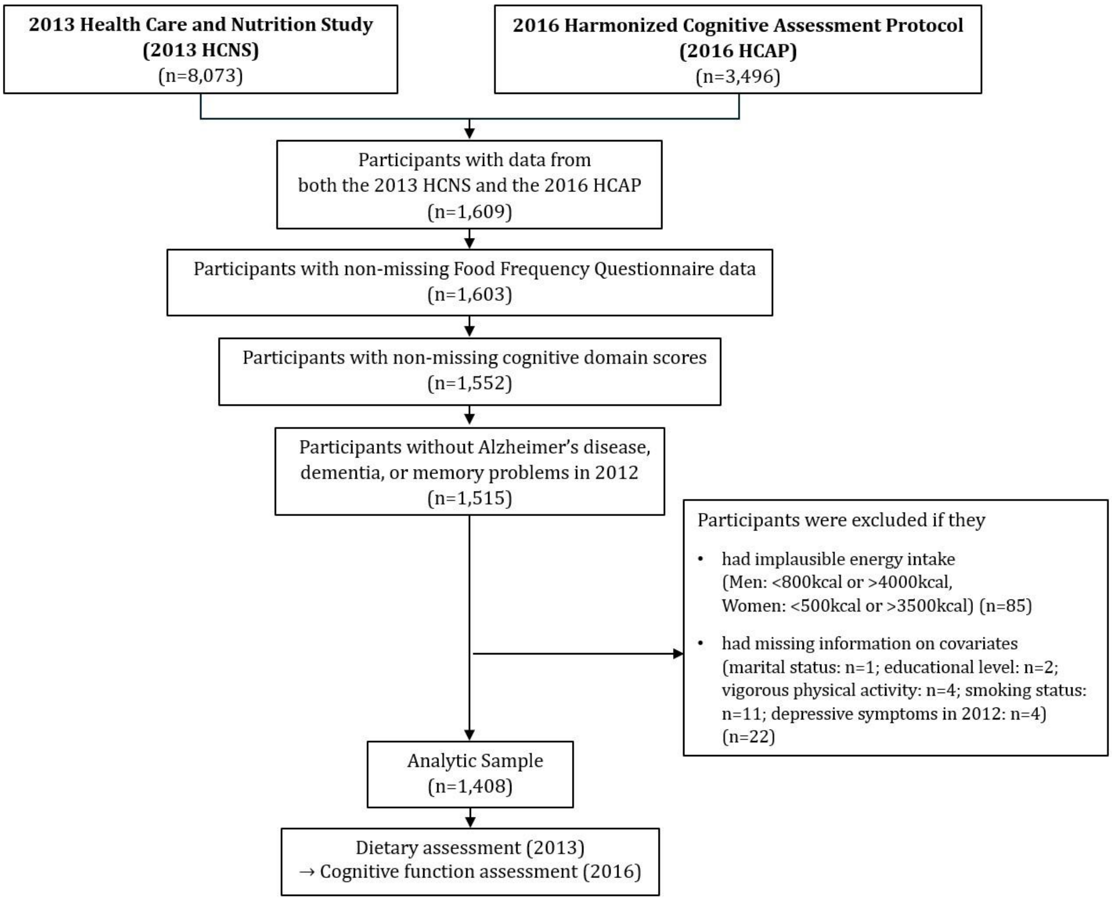


**Supplementary Figure 1. Flowchart of Health and Retirement Study (HRS) participants included in the analyses of UPF consumption and cognitive domain associations**

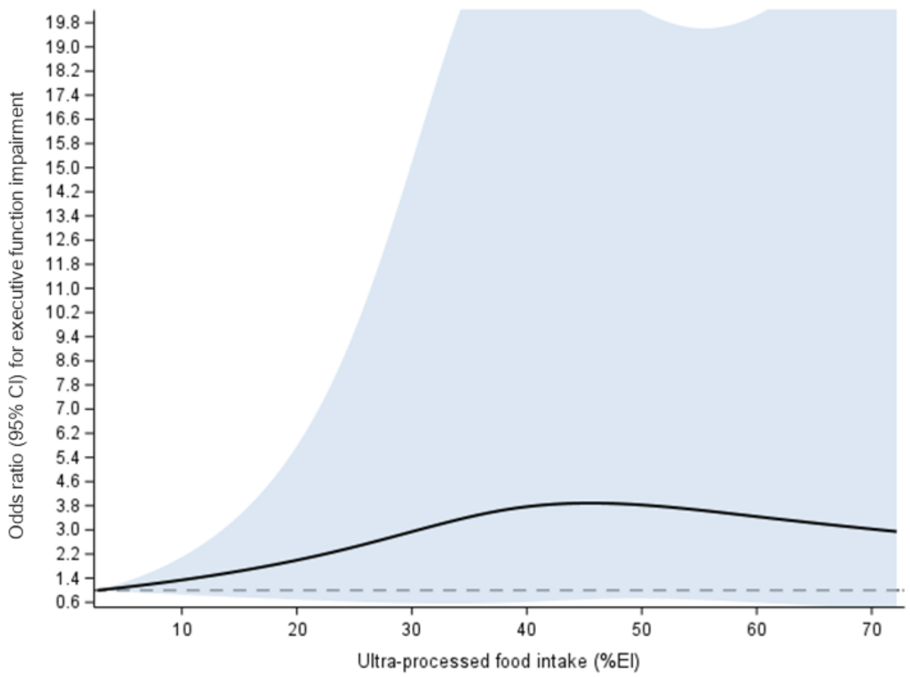


**Supplementary Figure 2. Restricted cubic spline curve showing the association between UPF intake and executive function impairment**

Solid line represents odds ratios (ORs); shaded area represents 95% confidence intervals (95% CIs).

## 2 Supplementary Tables

**Supplementary Table 1. Food items classified as ultra-processed foods (UPFs)**

| No. | Ultra-processed food subgroups | Food items included |
|---|---|---|
| 1 | **Whole grains** | Whole wheat/oatmeal/other whole grain bread |
| 2 | **Grains and derivatives** | Cold breakfast cereal; white bread (slice), including pita bread; rye or pumpernickel bread; bagels, English muffins, or rolls; breakfast bars (e.g., Nutrigrain, granola, Kashi) |
| 3 | **Dairy** | Regular ice cream; flavored yogurt, sweetened with fruit or other flavoring; frozen yogurt, sherbert or low-fat ice cream; cream cheese; yogurt, low carb, artificially sweetened or plain |
| 4 | **Fats and oils** | spreadable butter-butter/oil blend, added to food or bread exclude use in cooking; margarine or spread (small pat or tsp.), added to food or bread exclude us in cooking |
| 5 | **Processed meats** | bacon; beef/pork hot dog; chicken or turkey hot dogs or sausage; other processed meats (e.g., sausage, kielbasa, etc.); salami, bologna, or other processed meat sandwiches; hamburger lean; hamburger regular; |
| 6 | **Processed fish** | breaded fish cakes, pieces, or fish sticks |
| 7 | **Snacks and sweets** | candy bars (e.g., Snickers, milky way, Reese's); candy without chocolate (e.g., 1 pack mints, lifesavers); milk chocolate (e.g., Hershey's, M&Ms); dark chocolate (e.g., Hershey's dark or dove dark); cookies, fat free or reduced fat; cookies, other ready-made; brownies; doughnuts; cake, ready-made; pie, ready-made; energy bar; low carb bars (e.g., Atkins, Zone, South Beach); crackers, regular or low fat; other crackers; crackers, whole wheat or whole grain, e.g., Triscuits; jams, jellies, preserves, syrup, or honey; sweet roll, coffee cake or other pastry, fat free or reduced fat; sweet roll, coffee cake or other ready-made pastry; muffins or biscuits; fat free or light popcorn |
| 8 | **Sweeteners** | Splenda; other artificial sweetener |
| 9 | **Sugar-sweetened beverages** | carbonated beverage with caffeine and sugar (e.g., Coke, Pepsi, Mt. Dew, Dr. Pepper); other carbonated beverages with sugar (e.g., 7-up, Root Beer, Ginger Ale); other sugared beverages: punch, lemonade, sports drinks, or sugared iced tea; dairy coffee drink (hot/cold) (e.g., cappuccino); non-dairy coffee whitener; |
| 10 | **Other beverages** | Low-calorie beverage with caffeine, Diet coke, Diet Mt. Dew; Other low-calorie beverages without caffeine, Diet 7-up; soy milk |
| 11 | **Alcoholic beverages** | liquor (e.g., whiskey, gin, etc.) |
| 12 | **Sauces** | Ketchup or red chili sauce; low fat or fat free mayonnaise; regular mayonnaise; salad dressing; salsa, picante or taco sauce; applesauce |
| 13 | **Mixed dish** | Chowder or cream soup; French fried potatoes; pizza |

## Supplementary Table 2. Food items classified as unprocessed and minimally processed food

| No. | Unprocessed and minimally processed food subgroups | Food items included |
|---|---|---|
| 1 | **Whole grain** | Cooked oatmeal/cooked oat bran; brown rice; wheat germ |
| 2 | **Grains and derivatives** | Oat bran, added to food; other bran (wheat, etc.), added to food; white rice; other grains (e.g., bulgar, kasha, buckwheat, etc.); other cooked breakfast cereal |
| 3 | **Cereals** | Corn |
| 4 | **Tubers** | Yams or sweet potatoes; potatoes, baked, boiled, or mashed |
| 5 | **Vegetables** | Broccoli; cabbage or coleslaw; Brussel sprouts; spinach, cooked; spinach, raw; kale, mustard, or chard greens; iceberg or head lettuce; romaine or leaf lettuce; cauliflower; tomatoes; carrots raw; carrots cooked; mixed vegetables; orange squash; eggplant, zucchini or other summer squash; celery; peppers, green, yellow or red; onions as a garnish or in salad; onions as cooked vegetable, rings or soups; garlic |
| 6 | **Fruits and derivatives** | Raisins/grapes; prunes or dried plums; bananas; melon (cantaloupe, honeydew, watermelon); avocado; fresh apple or pears; oranges; grapefruit; strawberries, fresh, frozen or canned; other berries (e.g., blueberries, raspberries, blackberries) fresh, frozen or canned); peach/plum; apricot |
| 7 | **Dairy** | Skim milk; 1% or 2% milk; whole milk |
| 8 | **Meat** | Beef, pork, or lamb as a sandwich or mixed dish (e.g., stew, casserole, lasagna, frozen dinner, etc.); pork as a main dish (e.g., ham, or chops); beef or lamb as a main dish (e.g. steak, roast); liver (beef, calf, or beef); chicken or turkey sandwich or frozen dinner; other chicken or turkey, with skin-including ground; other chicken or turkey, including ground without skin; liver (chicken or turkey) |
| 9 | **Fish** | Shrimp, lobster, scallops, clams as a main dish; dark meat fish (e.g., tuna steak, mackerel, salmon, sardines, bluefish, swordfish); other fish (e.g., cod, haddock, halibut) |
| 10 | **Eggs and products** | Egg beaters or egg whites only; omega-3 fortified eggs, including yolk; regular eggs, with yolk |
| 11 | **Nuts and legumes** | Greem beans or string beans; peas or lima beans; beans or lentils, baked dried or soup; peanuts; walnuts; other nuts |
| 12 | **Snacks and sweets** | Pancakes or waffles; home baked cookies; home baked cake; homemade pie; home baked sweet roll, coffee cake or other pastry |
| 13 | **Non-alcoholic beverages** | Herbal tea or decaffeinated tea; decaffeinated coffee; calcium or vitamin D fortified orange juice; regular (not fortified) orange juice; grapefruit juice; other fruit juice (grape, cranberry); prune juice; water, bottled, sparkling or tap |
| 14 | **Caffeinated beverages** | Coffee with caffeine; tea with caffeine, including green tea |
| 15 | **Mixed dish** | Pasta (e.g., spaghetti, noodles, couscous, etc.) |

**Supplementary Table 3. Assessment of multicollinearity among covariates using variance inflation factors (VIFs)**

| Variable | Variance inflation factors (VIFs) |
|---|---|
| Age | 1.22 |
| Sex | 1.21 |
| Race and/or ethnicity | 1.03–1.19 |
| Marital status | 2.26 |
| Total net worth | 1.54–1.97 |
| Household size | 1.24–2.11 |
| Education | 1.26–1.50 |
| Smoking status | 1.15–1.18 |
| Alcohol consumption | 1.27–1.45 |
| Vigorous physical activity | 1.28–1.40 |
| Depressive symptoms at baseline | 1.07 |

VIFs were estimated using linear regression with executive function score as the dependent variable. Covariates with more than three categorical levels were included in the model as indicator (dummy) variables, and the range of VIFs across categories was reported.

**Supplementary Table 4. Top ten food groups contributing to energy intake from ultra-processed foods**

| Ranks | Total | Male | Female |
|---|---|---|---|
| 1 | Snacks and sweets (29.69%) | Snacks and sweets (30.03%) | Snacks and sweets (29.42%) |
| 2 | Grains and derivatives (14.67%) | Grains and derivatives (15.32%) | Grains and derivatives (14.15%) |
| 3 | Dairy (11.67%) | Processed meats (11.92%) | Dairy (13.12%) |
| 4 | Processed meats (11.05%) | Dairy (9.81%) | Processed meats (10.36%) |
| 5 | Sugar-sweetened beverages (9.29%) | Sugar-sweetened beverages (9.58%) | Sugar-sweetened beverages (9.06%) |
| 6 | Mixed dish (6.60%) | Mixed dish (6.58%) | Mixed dish (6.61%) |
| 7 | Sauces (5.90%) | Whole grains (5.34%) | Sauces (6.49%) |
| 8 | Whole grains (5.46%) | Sauces (5.13%) | Whole grains (5.56%) |
| 9 | Fats and Oils (2.55%) | Fats and Oils (2.38%) | Fats and Oils (2.68%) |
| 10 | Liquor (1.34%) | Liquor (2.20%) | Other beverages (0.91%) |

Values are presented as percent of energy intake derived from ultra-processed foods

**Supplementary Table 5. Multivariate-adjusted associations between percentage of energy intake from ultra-processed food (UPF) and domain-specific cognitive impairment additionally adjusted for total energy intake and body mass index**

| | Quintile 1 | Quintile 2 | Quintile 3 | Quintile 4 | Quintile 5 | P-trend |
|---|---|---|---|---|---|---|
| Executive function impairment | | | | | | |
| Cases/total | 24/281 | 37/282 | 23/282 | 46/282 | 33/281 | |
| OR (95% CI) | 1.00 | 1.68 (0.84-3.38) | 0.78 (0.41-1.46) | 2.18 (1.21-3.93) | 1.80 (1.01-3.20) | 0.036 |
| Memory impairment | | | | | | |
| Cases/total | 27/281 | 44/282 | 36/282 | 33/282 | 27/281 | |
| OR (95% CI) | 1.00 | 1.75 (0.82-3.76) | 1.11 (0.49-2.48) | 1.02 (0.50-2.09) | 0.63 (0.28-1.44) | 0.077 |
| Language impairment | | | | | | |
| Cases/total | 28/281 | 26/282 | 38/282 | 34/282 | 26/281 | |
| OR (95% CI) | 1.00 | 1.05 (0.41-2.69) | 2.14 (0.997-4.61) | 1.40 (0.67-2.90) | 1.20 (0.54-2.66) | 0.566 |
| Visuospatial impairment | | | | | | |
| Cases/total | 23/281 | 29/282 | 30/282 | 27/282 | 22/281 | |
| OR (95% CI) | 1.00 | 1.17 (0.57-2.42) | 1.10 (0.44-2.77) | 1.02 (0.43-2.39) | 0.72 (0.31-1.70) | 0.438 |
| Orientation impairment | | | | | | |
| Cases/total | 29/281 | 34/282 | 31/282 | 26/282 | 31/281 | |
| OR (95% CI) | 1.00 | 1.72 (0.87-3.40) | 1.07 (0.62-1.82) | 1.21 (0.51-2.89) | 2.17 (0.98-4.81) | 0.148 |

Sample size (n)= 1,408. Estimates are presented as odds ratios (ORs) and corresponding 95% confidence intervals (CIs).

[b]Models were adjusted for age (continuous, years), gender (men, women), and race/ethnicity (White, Black, Hispanic, and other), marital status (never married, married but spouse absent, separated, divorced, widowed; married or living with a partner), education (less than high school, high school graduate, some college/college graduate, post-college), total net worth (tertile), household size (1, 2, ≥3 members), vigorous activity (no, ≤once/week, >once/week), smoking (never smoker, ever smoker, current smoker), alcohol consumption (nondrinker, <5g/day, ≥5g/day), baseline depressive symptom (yes, no; CESD-8 score ≥5), total energy intake (kcal/day), and body mass index (<25, 25-30, ≥30 kg/m$^2$). All models accounted for the complex sampling design.

**Supplementary Table 6. Multivariate-adjusted associations between energy-adjusted ultra-processed food (UPF) intake and domain-specific cognitive impairment**

| | Quintile 1 | Quintile 2 | Quintile 3 | Quintile 4 | Quintile 5 | P-trend |
|---|---|---|---|---|---|---|
| Median (IQR), g/day | 216.8 (152.2-260.1) | 339.5 (313.9-359.6) | 426.0 (403.3-446.9) | 556.4 (515.2-614.3) | 888.3 (737.3-1181.0) | |
| Executive function impairment | | | | | | |
| Cases/total | 22/281 | 36/282 | 41/282 | 22/282 | 42/281 | |
| Model 1[b] | 1.00 | 1.98 (0.999-3.92) | 2.98 (1.51-5.88) | 1.49 (0.76-2.92) | 2.62 (1.28-5.35) | 0.043 |
| Model 2[c] | 1.00 | 1.96 (0.93-4.16) | 2.75 (1.29-5.85) | 1.36 (0.69-2.70) | 2.25 (1.06-4.80) | 0.172 |
| Memory impairment | | | | | | |
| Cases/total | 22/281 | 44/282 | 46/282 | 30/282 | 25/281 | |
| Model 1[b] | 1.00 | 2.42 (1.17-5.03) | 2.43 (1.34-4.42) | 1.67 (0.84-3.34) | 1.87 (0.90-3.90) | 0.611 |
| Model 2[c] | 1.00 | 2.07 (0.96-4.46) | 2.17 (1.13-4.14) | 1.45 (0.70-3.01) | 1.54 (0.73-3.25) | 0.958 |
| Language impairment | | | | | | |
| Cases/total | 26/281 | 31/282 | 28/282 | 33/282 | 34/281 | |
| Model 1[b] | 1.00 | 1.27 (0.64-2.51) | 1.38 (0.75-2.55) | 1.40 (0.68-2.90) | 1.60 (0.86-2.97) | 0.130 |
| Model 2[c] | 1.00 | 1.07 (0.56-2.05) | 1.12 (0.62-2.02) | 1.12 (0.54-2.33) | 1.19 (0.63-2.24) | 0.630 |
| Visuospatial impairment | | | | | | |
| Cases/total | 21/281 | 29/282 | 36/282 | 24/282 | 21/281 | |
| Model 1[b] | 1.00 | 1.01 (0.45-2.25) | 1.55 (0.73-3.26) | 0.59 (0.25-1.40) | 0.70 (0.30-1.65) | 0.183 |
| Model 2[c] | 1.00 | 1.02 (0.46-2.28) | 1.52 (0.68-3.39) | 0.61 (0.25-1.49) | 0.64 (0.26-1.61) | 0.139 |
| Orientation impairment | | | | | | |
| Cases/total | 27/281 | 28/282 | 38/282 | 29/282 | 29/281 | |
| Model 1[b] | 1.00 | 1.31 (0.62-2.79) | 1.45 (0.74-2.81) | 1.88 (0.96-3.66) | 2.59 (1.25-5.36) | 0.008 |
| Model 2[c] | 1.00 | 1.20 (0.52-2.77) | 1.29 (0.70-2.38) | 1.70 (0.90-3.21) | 2.23 (1.10-4.52) | 0.021 |

Sample size (n)= 1,408. Estimates are presented as odds ratios (ORs) and corresponding 95% confidence intervals (CIs).

Energy-adjusted UPF intake (g/day) was calculated using the residual method and categorized into sex-specific quintiles.

[b]Models were adjusted for age (continuous, years), gender (men, women), and race/ethnicity (White, Black, Hispanic, and other).

[c]Models were further adjusted for marital status (never married, married but spouse absent, separated, divorced, widowed; married or living with a partner), education (less than high school, high school graduate, some college/college graduate, post-college), total net worth (tertile), household size (1, 2, ≥3 members), vigorous activity (no, ≤once/week, >once/week), smoking (never smoker, ever smoker, current smoker), alcohol consumption (nondrinker, <5g/day, ≥5g/day), and baseline depressive symptom (yes, no; CESD-8 score ≥5). All models accounted for the complex sampling design.

**Supplementary Table 7. Multivariate-adjusted associations between percentage of energy intake from unprocessed or minimally processed foods and domain-specific cognitive impairment**

| | Quintile 1 | Quintile 2 | Quintile 3 | Quintile 4 | Quintile 5 | P-trend |
|---|---|---|---|---|---|---|
| Median (IQR), %EN | 29.5 (24.7-32.2) | 37.7 (35.9-39.4) | 43.3 (41.9-45.2) | 49.4 (47.8-50.9) | 58.8 (55.5-63.7) | |
| Executive function impairment | | | | | | |
| Cases/total | 37/281 | 33/282 | 38/282 | 28/282 | 27/281 | |
| Model 1[b] | 1.00 | 0.58 (0.35-0.96) | 0.72 (0.43-1.19) | 0.52 (0.27-1.01) | 0.47 (0.23-0.96) | 0.024 |
| Model 2[c] | 1.00 | 0.56 (0.33-0.93) | 0.73 (0.44-1.24) | 0.51 (0.25-1.01) | 0.42 (0.21-0.84) | 0.013 |
| Memory impairment | | | | | | |
| Cases/total | 23/281 | 30/282 | 38/282 | 38/282 | 38/281 | |
| Model 1[b] | 1.00 | 1.24 (0.64-2.40) | 1.50 (0.72-3.12) | 1.76 (0.86-3.59) | 1.63 (0.93-2.87) | 0.046 |
| Model 2[c] | 1.00 | 1.41 (0.69-2.90) | 1.59 (0.69-3.70) | 1.79 (0.81-3.94) | 1.61 (0.83-3.11) | 0.133 |
| Language impairment | | | | | | |
| Cases/total | 33/281 | 24/282 | 40/282 | 31/282 | 24/281 | |
| Model 1[b] | 1.00 | 0.72 (0.35-1.47) | 1.28 (0.60-2.74) | 1.00 (0.50-1.97) | 0.62 (0.29-1.33) | 0.424 |
| Model 2[c] | 1.00 | 0.81 (0.39-1.69) | 1.54 (0.69-3.42) | 1.22 (0.62-2.39) | 0.72 (0.33-1.59) | 0.751 |
| Visuospatial impairment | | | | | | |
| Cases/total | 31/281 | 21/282 | 25/282 | 30/282 | 24/281 | |
| Model 1[b] | 1.00 | 0.44 (0.21-0.92) | 0.50 (0.26-0.97) | 0.66 (0.38-1.15) | 0.63 (0.31-1.29) | 0.364 |
| Model 2[c] | 1.00 | 0.43 (0.20-0.90) | 0.55 (0.28-1.09) | 0.70 (0.40-1.21) | 0.64 (0.32-1.30) | 0.403 |
| Orientation impairment | | | | | | |
| Cases/total | 29/281 | 24/282 | 34/282 | 34/282 | 30/281 | |
| Model 1[b] | 1.00 | 0.51 (0.23-1.10) | 0.64 (0.29-1.40) | 0.55 (0.28-1.09) | 0.43 (0.19-0.97) | 0.051 |
| Model 2[c] | 1.00 | 0.48 (0.21-1.09) | 0.64 (0.28-1.44) | 0.60 (0.29-1.23) | 0.47 (0.19-1.15) | 0.145 |

Sample size (n)= 1,408. Estimates are presented as odds ratios (ORs) and corresponding 95% confidence intervals (CIs).

Energy-adjusted UPF intake (g/day) was calculated using the residual method and categorized into sex-specific quintiles.

[b]Models were adjusted for age (continuous, years), gender (men, women), and race/ethnicity (White, Black, Hispanic, and other).

[c]Models were further adjusted for marital status (never married, married but spouse absent, separated, divorced, widowed; married or living with a partner), education (less than high school, high school graduate, some college/college graduate, post-college), total net worth (tertile), household size (1, 2, ≥3 members), vigorous activity (no, ≤once/week, >once/week), smoking (never smoker, ever smoker, current smoker), alcohol consumption (nondrinker, <5g/day, ≥5g/day), and baseline depressive symptom (yes, no; CESD-8 score ≥5). All models accounted for the complex sampling design.
